# When SOREMPs Meet Episodic Hypersomnia: REM Dysregulation as a Trait or State in Kleine–Levin Syndrome

**DOI:** 10.1002/npr2.70186

**Published:** 2026-11-08

**Authors:** Shinichiro M. Koga

**Affiliations:** ^1^ Section for Pediatrics and Adolescent Medicine Japan Women's University Tokyo Japan; ^2^ Section for General Internal Medicine and Primary Care Tokyo University of Foreign Studies Tokyo Japan; ^3^ Department of Medicine, Tokyo Metropolitan Center for Cancer and Infectious Diseases Komagome Hospital Tokyo Japan

**Keywords:** central disorders of hypersomnolence, hypersomnia, Kleine‐Levin syndrome, multiple sleep latency test (MSLT), narcolepsy, REM sleep without atonia, sleep‐onset REM period (SOREMP)

## Abstract

REM dysregulation in Kleine–Levin syndrome may reflect a trait, an episode‐dependent state, or profound hypersomnolence; SOREMPs should therefore be interpreted in the context of the longitudinal clinical phenotype.

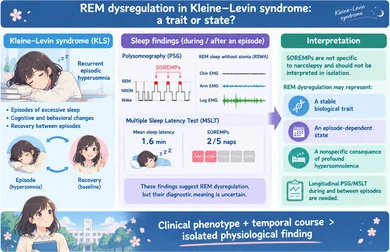

Dear Editor,

We read with great interest the report by Thawongit et al. describing a 12‐year‐old girl with Kleine‐Levin syndrome (KLS), in whom central hypopneas and REM sleep without atonia (RSWA) emerged during an episode and subsequently resolved following clarithromycin treatment [1]. The report provides valuable objective observations in a disorder for which polysomnographic data remain limited. We would nevertheless like to highlight an important implication of their case: the coexistence of a distinctly episodic clinical phenotype with a multiple sleep latency test (MSLT) showing two sleep‐onset REM periods (SOREMPs).

In the reported patient, the mean sleep latency was only 1.6 min and two SOREMPs were observed on MSLT [1]. These findings resemble the physiological abnormalities used in the evaluation of narcolepsy. However, MSLT findings should not be interpreted in isolation: narcolepsy Type 1 (NT1) requires compatible clinical or cerebrospinal fluid evidence, whereas narcolepsy Type 2 (NT2) requires exclusion of alternative causes of hypersomnolence [2, 3, 4]. KLS itself is characterized by recurrent, prolonged episodes with substantial recovery between episodes. Thus, the electrophysiological phenotype and longitudinal clinical phenotype need not map onto the same diagnostic category.

Importantly, SOREMPs are not exclusive to narcolepsy. Huang et al. reported that seven of 17 patients with KLS exhibited two or more SOREMPs during symptomatic periods, and MSLT findings varied according to the timing of assessment during the episode [2]. The diagnostic meaning of SOREMPs may depend substantially on the temporal and clinical context in which they are observed.

This issue also illustrates the broader difficulty of classifying central disorders of hypersomnolence [3, 4, 5, 6]. Rassu et al. [4] recently reported substantial overlap in total sleep duration and SOREMP frequency between idiopathic hypersomnia (IH) and NT2, despite differences in other REM‐related measures. Moreover, Cavalli et al. found diagnostic instability in NT2 and limited retest reliability of MSLT findings in NT2 and IH [5]. These observations suggest that a single physiological measurement may not fully capture the longitudinal phenotype of hypersomnolence disorders.

At the same time, the present case cannot establish that KLS alone accounts for the posttreatment MSLT result. The MSLT was performed after treatment initiation and was therefore not necessarily obtained under drug‐free conditions. Furthermore, a mean sleep latency of 1.6 min outside an overt episode leaves open the possibility of persistent hypersomnolence, insufficient sleep, or a coexisting sleep disorder. These possibilities cannot be excluded from a single case. Accordingly, the posttreatment MSLT should be regarded as an important observation rather than definitive evidence of a persistent REM phenotype.

Similar caution applies to the reported RSWA. Thawongit et al. propose that the coexistence of RSWA and central hypopneas may reflect dysfunction of brainstem sleep networks [1]. This is hypothesis‐generating, but RSWA in a single patient cannot establish specificity for KLS, causality, or reproducibility. Moreover, because RSWA assessment depends on electromyographic signals, channel‐specific artifacts, electrode placement, or contact should be considered when interpreting an isolated finding. The apparent disappearance of RSWA and central hypopneas after clarithromycin likewise demonstrates temporal association rather than a proven pharmacological or mechanistic effect.

Taken together, these observations raise a broader question of whether REM dysregulation in KLS represents a stable biological trait, an episode‐dependent state, or a nonspecific consequence of profound hypersomnolence. Future studies should therefore integrate longitudinal clinical phenotyping with standardized PSG and MSLT obtained during and between episodes, ideally under clearly documented medication and sleep‐duration conditions. Such studies may clarify whether REM abnormalities track the KLS state itself or persist beyond symptomatic episodes.

The report by Thawongit et al. thus raises an important diagnostic and pathophysiological question: when electrophysiological findings resemble narcolepsy but the longitudinal phenotype is characteristically episodic, how much diagnostic weight should be assigned to SOREMPs relative to the clinical course? Addressing this question may help clarify the boundaries among KLS, narcolepsy, and other central disorders of hypersomnolence.

## Author Contributions

S.M.K.: conceptualization, literature review, analysis, writing – original draft, writing – review and editing. The author approved the final version of the manuscript.

## Funding

The author has nothing to report.

## Conflicts of Interest

The author declares no conflicts of interest.

## Data Availability

Data sharing is not applicable because no new datasets were generated or analyzed for this Letter.
